# Diagnosis and Treatment of Augmentation Syndrome Secondary to Ropinirole: A Case Report

**DOI:** 10.7759/cureus.91241

**Published:** 2025-08-29

**Authors:** Rey D Nuez Lopez, Casilda Balmaceda

**Affiliations:** 1 General Medicine, Universidad Tecnologica de Santiago (UTESA), Santiago de los Caballeros, DOM; 2 Neurology, College of Physicians and Surgeons, Columbia University, New York City, USA

**Keywords:** augmentation, diagnosis, dopamine agonist treatment, low serum level of vitamin d, s: restless leg syndrome, treatment

## Abstract

*Augmentation* refers to the exacerbation of restless legs syndrome (RLS) symptoms caused by prolonged use of dopaminergic medications. It is marked by an earlier onset of symptoms, spread of symptoms to the upper limbs, a shorter interval before symptoms reappear during rest, increased symptom severity, and a diminished response to medication, which may necessitate progressively higher doses and could potentially worsen the symptoms. In this patient, a 61-year-old Caucasian female with a 20-year history of RLS, treated with gabapentin 300 mg twice daily and ropinirole 0.25 mg three to four times per day, augmentation was observed, likely due to long-term treatment with the dopamine agonist ropinirole in combination with low vitamin D levels. This case highlights the importance of recognizing augmentation in patients on long-term dopaminergic therapy and considering contributory factors such as vitamin D deficiency. Early identification and individualized treatment adjustments are essential to improving outcomes.

## Introduction

Restless legs syndrome (RLS), or Willis-Ekbom disease, is a sensorimotor disorder characterized by an uncontrollable urge to move the legs and unpleasant sensations, with symptoms that predominantly present in the evening or at nighttime and interfere with sleep [1]. The disorder affects approximately 1%-15.3% of the adult population aged 20-79 worldwide, representing roughly 356 million people, according to Song et al. [2]. Of these, 57.89% were women. Around 1%-3% of people experience severe and frequent symptoms [2,3]. The regions most affected are Europe and the Americas, while Africa is the least affected [2].

Although various medications can be used to treat RLS, dopaminergic drugs such as levodopa-carbidopa and the dopamine agonists pramipexole, ropinirole, and rotigotine are generally the most effective in reducing symptoms. However, long-term use of these medications can result in a challenging complication known as augmentation [1,3].

Augmentation describes a worsening of RLS symptoms and is marked by earlier onset, daytime manifestations, spread of symptoms to the upper limbs, a shorter time until symptoms appear at rest, increased symptom severity, and reduced response to medication [1]. This necessitates progressively higher doses, with the risk of no response and increasing symptom intensity. A notable feature of augmentation is the paradoxical intensification of symptoms when the dose of dopamine agonists is increased [1,3].

A history of smoking, current smoking status, alexithymia, high RLS severity, older age, and asthma scores appear to be important risk factors for developing augmentation syndrome. Another well-recognized risk factor is low ferritin levels (<50 µg/L) [4,5]. Several pathological mechanisms, including dysfunction in dopamine-related systems, alterations in adenosine and glutamatergic pathways, brain iron deficiency, dopamine dysregulation, a hyperglutamatergic state, a hypoadenosinergic state, and genetic mutations, are likely involved in the development of RLS [1,5]. The effectiveness of drugs and therapies that target these mechanisms further underscores their crucial roles in the condition [3].

The long-term effectiveness of levodopa and other dopaminergic agonists has been well documented, making them the first-line pharmacological treatments for RLS in recent years [1]. However, even at low doses, prolonged use of these drugs results in a gradual worsening of RLS symptoms. Tolerance differs from augmentation in the sense that a higher dose of a drug is needed to produce the same effect, while in augmentation, the effects are produced by prolonged exposure to the drug, regardless of the dose. This makes augmentation the primary reason for treatment discontinuation and failure in RLS therapy [1,3].

## Case presentation

We present the case of a 61-year-old Caucasian female with a 20-year history of RLS, treated with gabapentin 300 mg twice daily and ropinirole 0.25 mg three to four times per day since her diagnosis. The patient presented to a neurology outpatient clinic with a chief complaint of spontaneous movements of the arms. She had been compliant with her medication regimen since her diagnosis.

The patient reported being stable until two weeks before her consultation, when she began to experience an urge to shake and jerk her arms spontaneously, without any consistent pattern or specific timing, and with no other associated symptoms. These movements led to sleep deprivation, with no apparent triggers. The only activities that alleviated these movements were those requiring concentration, such as playing games on her cell phone or doing housework. The patient’s vital signs were within the normal range, and no fever was detected.

On examination, the patient was alert and oriented to person, place, and time, but displayed an anxious mood. Palpation revealed no signs of stiffness or rigidity. During the examination, she exhibited several spontaneous upper limb movements, including rotary motions, thumb-pursuing gestures, and undulating movements. These movements ceased when she was distracted during the reflex assessment. Reflexes in both the upper and lower limbs were normal.

The patient underwent blood tests, including assessments for Lyme disease due to a recent tick bite and the sudden exacerbation of RLS with new neurological symptoms. Serum vitamin D levels and an iron panel consisting of total iron, iron saturation, transferrin, ferritin, and total iron-binding capacity (TIBC) were also measured. Her complete blood count (CBC), renal function tests, liver function tests, iron panel, and electrolytes were normal. Vitamin D levels were low (19.4 ng/dL), consistent with deficiency (Table 1). Lyme disease tests were negative (Tables 2-3). A magnetic resonance imaging (MRI) performed before her consultation to rule out an anatomically visible neoplasm was reported as normal, and the electroencephalogram (EEG) was also normal.

**Table 1:** Laboratory test results. WBC, white blood cells; RBC, red blood cells; HGB, hemoglobin; HCT, hematocrit; MCV, mean corpuscular volume; MCHC, mean corpuscular hemoglobin concentration; MCH, mean corpuscular hemoglobin; PLT, platelets; ALT, alanine aminotransferase; AST, aspartate aminotransferase; ALP, alkaline phosphatase; BUN, blood urea nitrogen; eGFR, estimated glomerular filtration rate; HCO_3_, bicarbonate; TIBC, total iron-binding capacity

Blood test	Values	Reference values
WBC	7.9 x 10^9^/L	3.6 x 10^9^-11.0 x 10^9^/L
RBC	4.56 x 10^12^/L	3.80 x 10^12^-5.20 x 10^12^/L
HGB	13.8 g/dL	11.9-16.0 g/dL
HTC	42.00%	35%-47%
MCV	92 fL	75-100 fL
MCHC	32.9 g/dL	32.0-35.0 g/dL
MCH	30.3 pg	26.0-33.0 pg
PLT	261 x 10^9^/L	140 x 10^9^-440 x 10^9^/L
ALT	11 U/L	5-33 U/L
AST	15 U/L	9-40 U/L
ALP	94 U/L	30-121 U/L
BUN	25 mg/dL	8-23 mg/dL
Creatinine	0.83 mg/dL	0.51-1.15 mg/dL
eGFR	80 mL/min/1.73 m^2^	>59 mL/min/1.73 m^2^
Sodium in serum	142 mmol/L	135-148 mmol/L
Potassium in serum	4.7 mm/L	3.5-5.5 mmol/L
Chloride in serum	106 mmol/L	96-107 mmol/L
Calcium in serum	10 mg/dL	8.6-10.5 mg/dL
Magnesium in serum	2.1 mg/dL	1.6-2.4 mg/dL
HCO3	20 mmol/L	18-32 mmol/L
Glucose	82 mg/dL	70-100 mg/dL
Ferritin	76 ng/mL	13-150 ng/mL
TBIC	314 ug/dL	149-492 ug/dL
Transferrin	265 mg/dL	204-360 mg/dL
Iron	68 ug/dL	60-160 ug/dL
Iron saturation	22%	15%-45%
Vitamin D levels	19.4 ng/dL	21-29 ng/dL

**Table 2:** Lyme IgG antibody by line blot, serum. Lyme IgG line blot interpretation Positive: Five or more of the following Borrelia-specific bands: 18, 23, 28, 30, 39, 41, 45, 58, 66, and 93. Negative: No bands, or banding patterns that do not meet the positive criteria. IgG, immunoglobulin G

Lyme Ab IgG by line blot	Current results	Reference values
IgG P93 Ab.	Absent	Absent
IgG P66 Ab.	Absent	Absent
IgG P58 Ab.	Absent	Absent
IgG P45 Ab.	Present	Absent
IgG P41 Ab^.^	Present	Absent
IgG P39 Ab.	Absent	Absent
IgG P30 Ab.	Present	Absent
IgG P28 Ab.	Absent	Absent
IgG P23 Ab.	Absent	Absent
IgG P18 Ab.	Absent	Absent
Lyme IgG line blot interp.	Negative	Negative

**Table 3:** Lyme IgM antibody by line blot, serum. Lyme IgM line blot interpretation Positive: Two of the following bands: 23, 39, or 41. Negative: No bands, or banding patterns that do not meet the positive criteria. IgM, immunoglobulin M

Lyme Ab IgM by line blot	Current results	Reference values
IgM P41 Ab.	Absent	Absent
IgM P39 Ab.	Absent	Absent
IgM P23 Ab.	Absent	Absent
Lyme IgM line blot interp.	Negative	Negative

Initially, the patient’s treatment was adjusted to ropinirole 1 mg two to three times daily to control RLS due to a high suspicion of tolerance, but this resulted in worsening of her symptoms after five days of use. To address her insomnia, zolpidem 10 mg before bedtime was prescribed.

Based on the findings above, Lyme disease was ruled out as the cause of the exacerbation and new onset of neurological symptoms. Augmentation syndrome due to prolonged use of ropinirole for RLS was considered the most likely cause of symptom worsening, potentially aggravated by the patient’s low vitamin D levels [1]. Dividing the doses of ropinirole into afternoon and evening administrations is recommended according to current guidelines [3]. Additionally, based on previous studies, such as those by Zeng et al., supplementation with vitamin D when levels are insufficient or deficient, or iron when ferritin levels are below 50 mcg/L, is recommended [1].

A gradual tapering of ropinirole was initiated, reducing the dose from 1 mg two to three times daily to 0.50 mg twice daily, and then further to 0.25 mg twice daily until discontinuation. Weekly supplementation with 1,200 IU of vitamin D was also started. For insomnia, the patient was initially treated with zolpidem 10 mg, but this was subsequently changed to clonazepam 1 mg due to lack of response. After this adjustment, the patient demonstrated significant improvement in spontaneous arm movements and sleep quality at follow-up after a couple of weeks.

## Discussion

Long-term use of dopamine agonists, including pramipexole, ropinirole, rotigotine, and levodopa, is a well-recognized cause of augmentation syndrome [6]. On average, dopaminergic drugs used in the long-term treatment of RLS can induce augmentation after approximately 2.7 ± 2.4 years of continuous pharmacotherapy [6]. This complication occurs in at least 20% of patients treated with dopamine receptor agonists and in up to 80% of those treated with levodopa [6].

Our patient experienced the spread of symptoms to her arms, along with improvement during physical activity. Notably, activities requiring intense concentration, such as playing strategy-based games, almost or completely alleviated her symptoms. The combination of these symptoms led to severe anxiety and insomnia, which, in turn, may serve as fertile ground for the development of other neuropsychiatric disorders [6]. Although most studies report augmentation syndrome as a complication of carbidopa-levodopa use, other dopaminergic agonists such as ropinirole can also induce this syndrome, as demonstrated in our patient, who had been using ropinirole for 20 years.

According to the Max Planck Institute (MPI) criteria (Table 4) [6-7], our patient met the following:

(A) Basic features: Increase in severity on several days of the week, no changes in medical status or lifestyle, and several years of good response to treatment.

(B) Persisting paradoxical response to treatment: Increase in the severity of RLS symptoms.

(C) Earlier onset of symptoms: Earlier onset of symptoms, spread to the upper limbs, and shorter relief from medication.

Together, these findings satisfy the diagnostic criteria for augmentation syndrome.

**Table 4:** Max Planck Institute (MPI) criteria: diagnostic criteria for augmentation syndrome. Augmentation requires criteria A + B or A + C or A + B + C to be met [6-7].

MPI criteria
A: Basic Features: The increase in symptom severity was experienced on five out of seven days during the previous week. The increase in symptom severity is not accounted for by other factors such as a change in medical status, lifestyle, or the natural progression of the disorder. It is assumed that there has been a prior positive response to treatment
B: Persisting (although not immediate) paradoxical response to treatment: RLS symptom severity increases sometimes after a dose increase and improves sometimes after a dose decrease.
C: Earlier onset of symptoms: An earlier onset by at least four hours. OR: An earlier onset (between two and four hours) occurs with one of the following compared to the symptom status before treatment Shorter latency to symptoms when at rest; spreading of symptoms to other body parts; intensity of symptoms is greater (or an increase in periodic limb movements (PLM) if measured by polysomnography (PSG) or the suggested immobilization test (SIT)); the duration of relief from treatment is shorter.

Laboratory tests ruled out Lyme disease as a differential diagnosis, despite the patient reporting a tick bite before symptom onset. However, the patient’s low vitamin D levels were concerning, as a previous case report demonstrated significant improvement in dopaminergic augmentation with vitamin D supplementation in an 81-year-old woman [1]. The patient’s iron levels and related blood tests were normal.

Our patient had a normal neurological examination aside from the movement disturbances in her arms. The primary issue was the arm shaking and jerking. Initially, we considered arm restlessness syndrome as a probable diagnosis and prescribed an increased dose of ropinirole 1 mg up to three times daily. However, when the patient’s symptoms worsened, further investigation was pursued. Since her clinical presentation met the criteria discussed earlier, augmentation syndrome secondary to ropinirole was suspected. During the interview, the patient’s described movements were confirmed, with slight exacerbation while speaking, though these symptoms were noticeably reduced when she was walking or distracted during examination. The physical examination showed no hyperreflexia, proprioceptive deficits, sensory changes, or muscle weakness.

It is important to note that an exacerbation of RLS and augmentation syndrome can share some clinical characteristics, making early diagnosis and differentiation crucial for the patient’s well-being. Prompt recognition can prevent the development of serious complications such as depression. According to Song et al., depression has been associated with an odds ratio (OR) of 1.71 (95% confidence interval (CI) = 1.26-2.32), and a significant portion of patients with concurrent RLS and depression have reported suicidal thoughts due to their RLS symptoms [2,3,8]. Given this, early diagnosis and appropriate treatment of augmentation syndrome in patients receiving dopamine agonists are essential. It is also necessary to assess for vitamin D deficiency or low iron levels, as these can exacerbate or even trigger symptoms.

Based on several investigations, the treatment plan for our patient included dividing the doses of ropinirole into morning/afternoon and night administrations [7,9-10]. Vitamin D supplementation at 1,200 IU weekly was initiated, as evidence suggests that low vitamin D levels can exacerbate augmentation syndrome [1]. Additionally, short-term clonazepam 1 mg was prescribed to help with insomnia and reduce arousal due to periodic limb movements during sleep, given the lack of response to zolpidem 10 mg and existing evidence supporting its use in such cases [3,11]. Gabapentin 300 mg was reintroduced, given its comparable efficacy to dopaminergic agents in managing RLS symptoms, as the patient had discontinued it on her own due to a perceived lack of benefit [3,6-11].

We initiated a gradual tapering of ropinirole from 1 mg (two to three times daily) to 0.50 mg twice daily, and then to 0.25 mg twice daily, with the goal of discontinuation based on the patient’s response. A potential switch to rotigotine was considered if adequate symptom control with gabapentin could not be achieved [6,7,9,12]. Following these changes, the patient reported improvement in her symptoms and better sleep quality.

## Conclusions

Augmentation syndrome remains one of the major challenges in the long-term dopaminergic treatment of RLS. Although RLS is a well-recognized disorder, augmentation continues to be an area that requires further investigation. Given that current evidence is limited to a few case reports, larger cohort studies are needed to clarify and strengthen this potential link. Effective management requires a patient-centered approach that considers nutritional deficiencies, genetic predispositions, and individualized medication strategies.
